# Accuracy evaluation of dental CBCT and scanned model registration method based on pulp horn mapping surface: an in vitro proof-of-concept

**DOI:** 10.1186/s12903-024-04565-3

**Published:** 2024-07-22

**Authors:** Dianhao Wu, Jingang Jiang, Jinke Wang, Shan Zhou, Kun Qian

**Affiliations:** 1 The Key Laboratory of Advanced Manufacturing and Intelligent Technology, Ministry of Education, Harbin University of Science and Technology, NO. 52, Xuefu Road, Nangang Dist, Harbin, Heilongjiang Province 150080 People’s Republic of China; 2 The Robotics and its Engineering Research Center, Harbin University of Science and Technology, Harbin, Heilongjiang Province 150080 China; 3 The 2nd Affiliated Hospital of Harbin Medical University, No.246 Xuefu Road, Nangang District, Harbin, Heilongjiang Province 150001 People’s Republic of China; 4 The Peking University School of Stomatology, No.22 Zhongguancun South Street, Haidian District, Beijing, 100081 People’s Republic of China

**Keywords:** Root canal therapy, Digital registration, CBCT, Scanned model, Crown-pulp pose deviation

## Abstract

**Background and aim:**

3D fusion model of cone-beam computed tomography (CBCT) and oral scanned data can be used for the accurate design of root canal access and guide plates in root canal therapy (RCT). However, the pose accuracy of the dental pulp and crown in data registration has not been investigated, which affects the precise implementation of clinical planning goals. We aimed to establish a novel registration method based on pulp horn mapping surface (PHMSR), to evaluate the accuracy of PHMSR versus traditional methods for crown-pulp registration of CBCT and oral scan data.

**Materials and methods:**

This vitro study collected 8 groups of oral scanned and CBCT data in which the left mandibular teeth were not missing, No. 35 and No. 36 teeth were selected as the target teeth. The CBCT and scanned model were processed to generate equivalent point clouds. For the PHMSR method, the similarity between the feature directions of the pulp horn and the surface normal vectors of the crown were used to determine the mapping points in the CBCT point cloud that have a great influence on the pulp pose. The small surface with adjustable parameters is reconstructed near the mapping point of the crown, and the new matching point pairs between the point and the mapping surface are searched. The sparse iterative closest point (ICP) algorithm is used to solve the new matching point pairs. Then, in the C +  + programming environment with a point cloud library (PCL), the PHMSR, the traditional sparse ICP, ICP, and coherent point drift (CPD) algorithms are used to register the point clouds under two different initial deviations. The root square mean error (RSME) of the crown, crown-pulp orientation deviation (CPOD), and position deviation (CPPD) were calculated to evaluate the registration accuracy. The significance between the groups was tested by a two-tailed paired *t*-test (*p* < 0.05).

**Results:**

The crown RSME values of the sparse ICP method (0.257), the ICP method (0.217), and the CPD method (0.209) were not significantly different from the PHMSR method (0.250). The CPOD and CPPD values of the sparse ICP method (4.089 and 0.133), the ICP method (1.787 and 0.700), and the CPD method (1.665 and 0.718) than for the PHMSR method, which suggests that the accuracy of crown-pulp registration is higher with the PHMSR method.

**Conclusion:**

Compared with the traditional method, the PHMSR method has a smaller crown-pulp registration accuracy and a clinically acceptable deviation range, these results support the use of PHMSR method instead of the traditional method for clinical planning of root canal therapy.

## Introduction

RCT controls the microbial infection in the root canal by proper shaping and disinfection of the root canal system to promote the healing of periapical lesions and tissue regeneration [1]. Physicians are required to have rich clinical experience, with the help of familiar anatomical knowledge, accurate 3D images, and stable operation, the exploration and preparation are carried out along the expected root canal access [2]. CBCT can provide 3D structural information on teeth, bones, main root canals, and periapical tissues [3]. Due to limited image resolution and various artifacts (including metal-induced artifacts), dental CBCT alone may not be able to describe the precise details of the tooth surface [4]. The CBCT and the optical surface scanned data were registered and integrated into the guided surgery software, which can express the crown and internal anatomical details at the same time [5]. Researchers have adopted the surgical method of “inlay-guided endodontics” by integrating CBCT and 3D scanned data, designing the best virtual pulp opening access, and accurately placing the file into the planned position with the help of a 3D printing guide plate [6–9]. Torres et al. have used 3D models for intraoperative dynamic navigation of RCT [10]. Compared with free hand operation, the use of the above digital methods can reduce the difference between the planned and actual instrument positions, accurately visualize its path and surrounding oral anatomy, and achieve low-risk, minimally invasive, and precise treatment. The registration process greatly affects the deviation of the designed virtual access or guide plate.

Commonly used clinical registration can be divided into two categories, marker-based registration is to fix the existing markers around the surgical area in a certain way, such as bone implant nails [11], occlusal splints [12], and Lego bricks [8], and the markers of the two scans can be aligned. However, this method takes up a certain amount of oral space and invasive operation will cause discomfort to patients. For unmarked registration, on the one hand, the anatomical features in the surgical area can be used as special markers, such as cusps [13], etc., but the method is not robust when tooth features are missing; On the other hand, using 3D point set registration algorithms, including point-based (e.g. ICP [14]) and surface-based registration, which are the computationally fast and simple method in oral clinical practice [15–19].

Currently, traditional algorithms embedded in Mimics, Rapidform, and Geomagic Studio software are mostly used for clinical registration. Kim et al. used manual surface-based registration, and the mean registration accuracy of mandibular teeth was 0.13 ± 0.11 mm [15]. Lim et al. used the three-point method for manual coarse registration, and then used the ICP algorithm for fine registration. The mean registration accuracy of the mandibular was 0.21 ± 0.04 mm [16]. Lee et al. developed a registration method for orthodontic treatment, using surface-based fine automatic registration. The difference between the expected and the actual maxillary root position was 0.1678 ± 0.3178 mm [17]. Ye et al. used the same method and reported that the registration errors of CBCT pixels at 0.2 mm and 0.4 mm were 0.163 ~ 0.231 mm and 0.202 ~ 0.345 mm, respectively [18]. Guo et al. used a manually operated three points structure reference plane for point-based registration, and the registration accuracy of the lower jaw was 0.151 ± 0.0337 mm [19]. Sun et al. used surface-based regional automatic registration with a mean registration error of 0.33 mm [20]. Yi et al. used the ICP algorithm to align the virtual registration unit and optical scanned data to evaluate the dental implant accuracy [21]. Rangel et al. placed titanium markers on the gingiva to match dental casts into CT data, and the mean deviation of the lower jaw were and 0.30 mm [22].

Some scholars have proposed other registration methods. Bai proposed to generate a multi-layer spherical point set inside the marker ball, and used the weighted ICP algorithm to perform multi-layer point set registration, which solved the problem of surface height deformity of the laser scanning marker ball, and the mean square error was 0.301 mm [23]. Yau et al. adopted the three-point location and ICP algorithm to achieve the crown registration [24]. Zhang et al. [25] used the principal component analysis and the ICP method to register the two models. Dai et al. [26] proposed a multi-point registration method to achieve rapid registration of crown optical scanned data and root CBCT data. Wang et al. [27] used the sparse ICP method to achieve accurate registration of different dentition coordinate systems. Lin et al. [28] proposed an improved iterative ICP algorithm and convex hull selection method for the automatic segmentation of dentition models. Lee et al. [5] proposed a novel depth map-based registration method to register 3D surface models, and performed the ICP method for the subsequent fine registration. Lee et al. proposed deep-pose regression neural networks and optimal clusters for automatic registration [29]. However, these recent studies only considered the registration deviation from crown to crown, and did not introduce pulp pose information into the registration. Moreover, the effectiveness of traditional methods for pulp to crown registration has not been studied.

The study aims to propose a registration method based on the pulp horn mapping surface (PHMSR). Four methods (PHMSR, sparse ICP [30], ICP [14], and CPD [31]) were used to register the CBCT and optical scanned point cloud of the left mandibular teeth. The RSME and crown-pulp pose deviations after registration were compared. The null hypothesis was that no difference would be found in the precision of the crown-pulp registration between the PHMSR and conventional methods.

### Materials and method

#### Study design

**Fig. 1:**
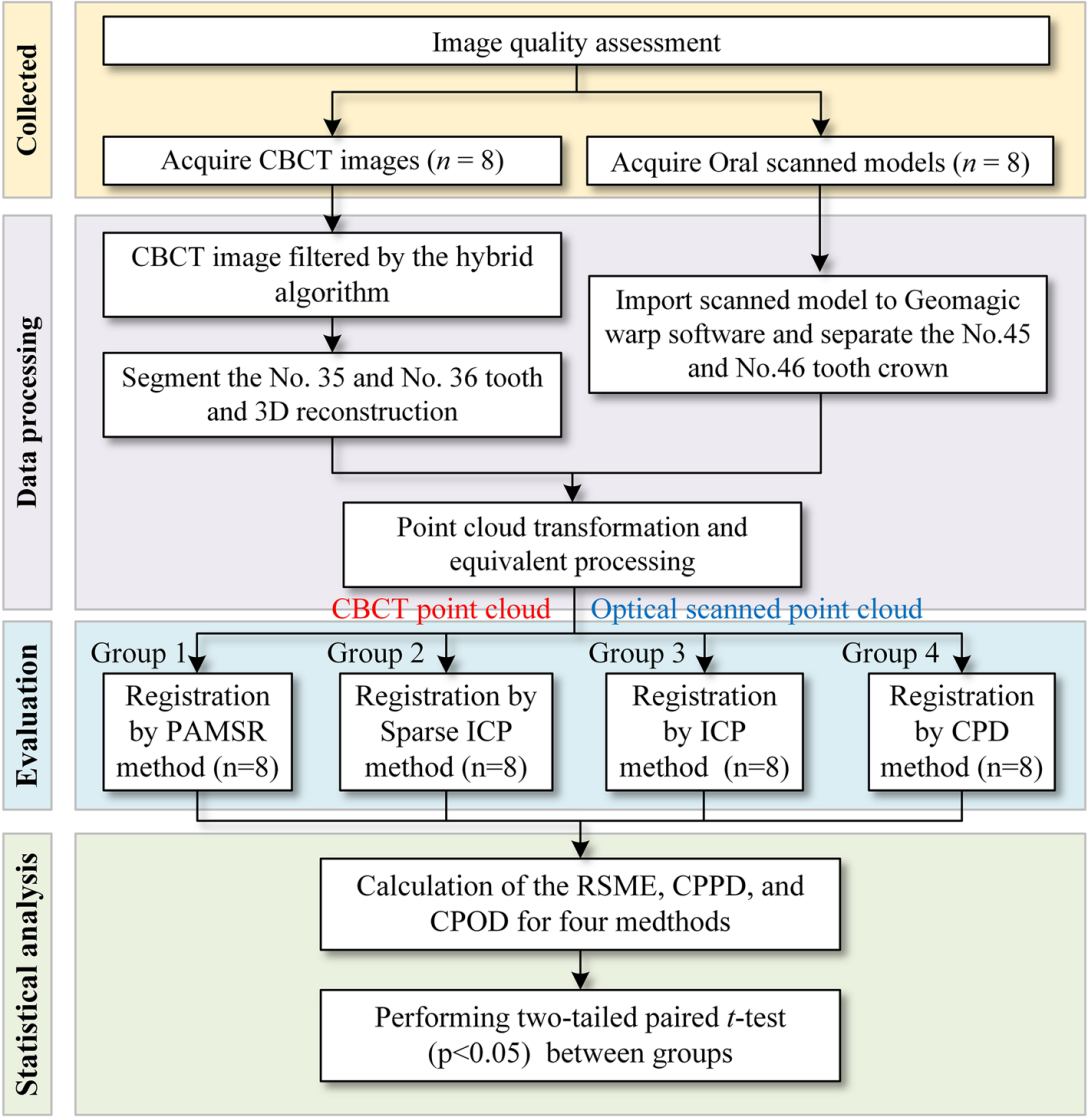
Research flow

This study was approved by the Medical Ethics Committee of the 2nd Affiliated Hospital of Harbin Medical University (YJSKY2022-115) and informed consents sought from the subjects whose scans were used as a study material. The sample size was calculated by a software program (G*power 3.1.9.7, Heinrich-Heine-Universität, Germany), assuming that the alpha error of 5%, a study power of 80%, and an effect size of 0.92, a minimum sample size of *n* = 8 per group was needed [32]. All oral scanned and CBCT data were selected from the 2nd Affiliated Hospital of Harbin Medical University, the subjects were aged between 25 and 35 years old, 4 males and 4 females. The inclusion criteria were no loss of left mandible teeth, no artifacts, and tooth crowns were not severely damaged. Exclusion criteria were no history of endodontic treatment or no anatomical variations to avoid artifacts hampering the registration of the real main anatomical configurations. To reduce the calibration error of the anatomical variation, intraobserver and interobserver calibrations were previously performed while examining selected sample images with diverse morphology. Each CBCT was evaluated and interpreted by two radiologists twice with a one-week interval between assessments. The reliability data was analyzed using the weighted Kappa test, and the Kappa coefficients of all samples were greater than 0.91. The study images were assessed independently. The research flow is shown in Fig. 1.

### Experimental equipment and software

Each CBCT was collected by an oral X-ray computed tomography system (SBR3D, SOREDEX PaloDEx Group Oy, Finland). The dimensions of CBCT are 580 × 580 × 300, and the resolution is 0.25 × 0.25 × 0.25 mm. All optical scanned data were collected by a digital intraoral scanner (iTero Element, Align Technology, USA), and the scanning accuracy was $20\mu m$. The number of point clouds is about 150000, forming 300000 triangular meshes.

We used Matlab 2020b (MathWorks, USA) for image filtering, segmentation, 3D reconstruction, and registration error calculation, and used reverse engineering software Geomagic warp 2017 (3D System, USA, Morrisville) for extraction of target teeth and processing of 3D point cloud. The PHMSR, ICP [14], sparse ICP [31], and coherent point drift (CPD) [32] algorithms were based on the C +  + programming language with the PCL. For experiment evaluation, the experiment environment is Ubuntu 20.04 with X64 processor (AMD Ryzen 5 3600 6-Core Processor). Statistical analyses were performed using SPSS software (version 22.0; IBM Corp., Armonk, NY, USA).

### Data processing

All CBCT data were exported to the Digital Imaging and Communications in Medicine (DICOM) format. All scanned data are exported to STL format. No. 35 and No. 36 teeth were selected as the target teeth.

**Fig. 2:**
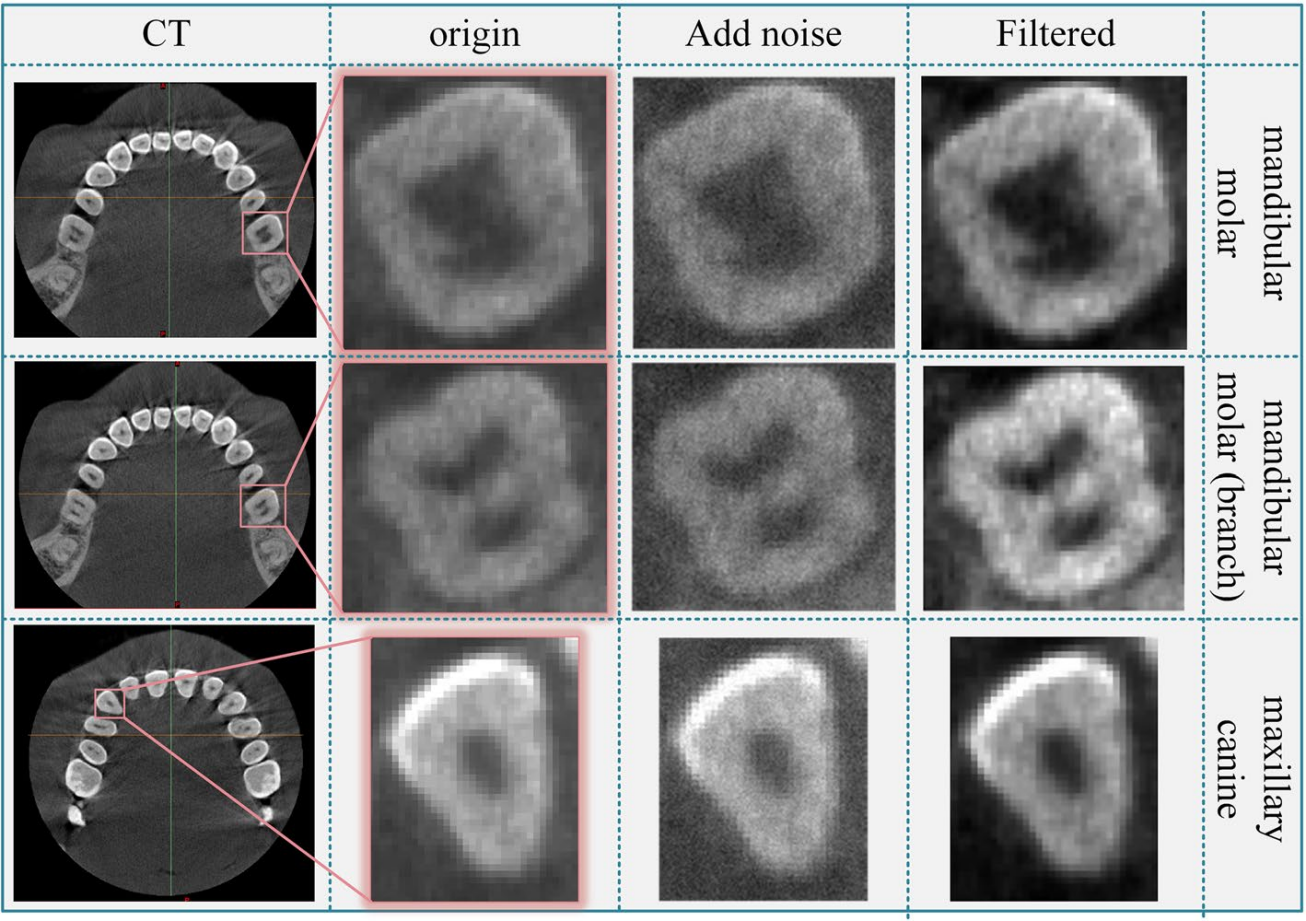
The Wavelet + NL-means hybrid filtering results

In general, the anatomical region boundary of the pulp cavity in the actual CBCT images are generally low in brightness, and blurred in the boundary. In the process of medical image acquisition, the unknown mixed noise will affect image quality, so the pulp cavity is difficult to capture. To minimize the effects of noise and enhance the strength of teeth and pulp contours, the wavelet [33] and NL-means [34] hybrid filtering algorithm is used to perform filtering operations on CBCT. We set the block size to 2, the search window to 5, and the decay parameter to 10 to obtain the filtered result shown in Fig. 2, the edges of the pulp and enamel were clearer in CBCT, and the contrast between enamel and alveolar bone was enhanced.

**Fig. 3:**
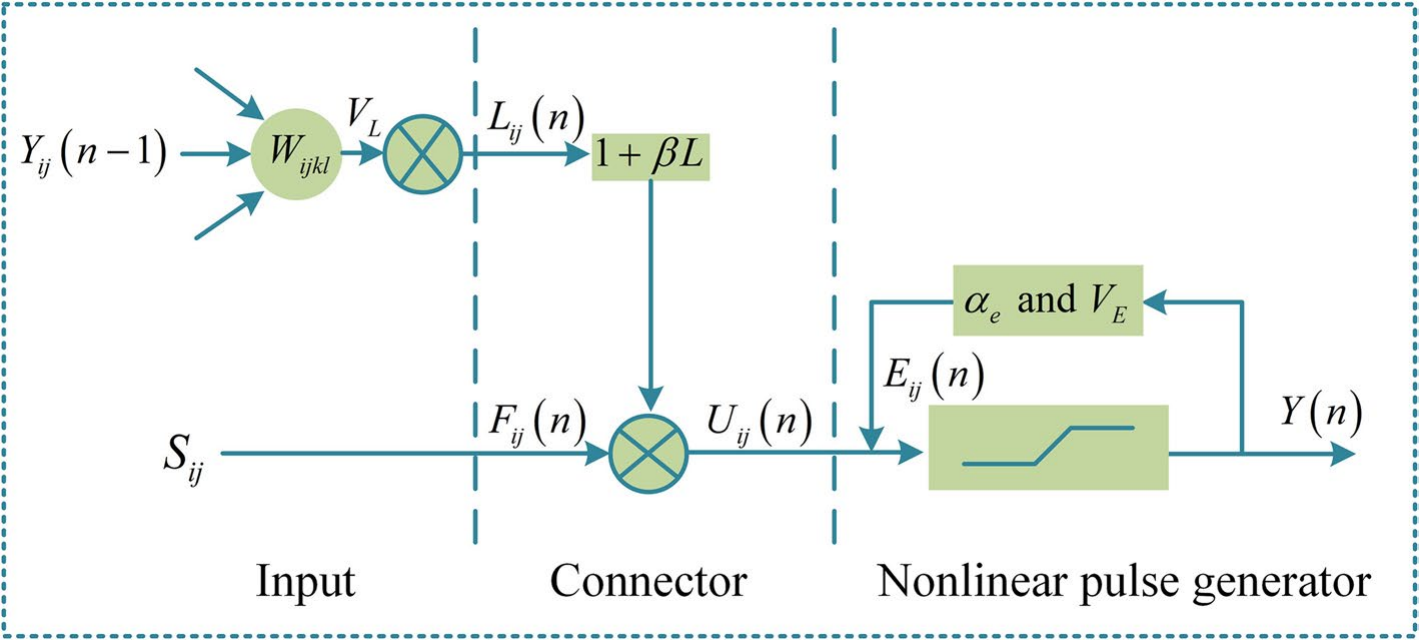
Working principle of the SCM model, $S_{ij}$ is external excitation, $F_{ij} \left( n \right)$ is the feedback input, $L_{ij} \left( n \right)$ is the coupling connection input, $U_{ij} \left( n \right)$ is the internal activity, $E_{ij} (n)$ is the dynamic threshold, $Y_{ij} (n)$ is pulse output, $\alpha_{e}$ is the amplitude of the $E_{ij} (n)$, $\beta$ is the connection strength, $V_{E}$ is the exponential decay time constant, $W_{ijkl}$ is the weight matrix for coupled connected domains, $V_{L}$ is the amplification factor of the coupled connection domain

The PCNN has many unique properties, including pulse coupling, pulse synchronization, multiplicative modulation, and variable thresholds, which has been used for the segmentation of dental images. Since there are too many parameters and the network is miscellaneous [35], we applied the spiking cortical model (SCM) [36] for individual tooth and pulp segmentation for CBCT, as shown in Fig. 3. Compared with the PCNN model, the SCM model simplifies the internal activity term and feedback input part, but it still maintains the feedback input and connection input. The internal activity in the SCM model is more closely related to the external excitation, which better captures the intensity changes in the pulp cavity. However, CBCT images with different spatial resolution, voxel size, and sharpness make it difficult to determine the size of their parameter values. To improve the segmentation accuracy and exclude the deviation of subjective factors, the adaptive parameter rules for $\alpha_{e}$, $V_{E}$, $\beta$ and $\alpha_{u}$ are designed as follows:

The value of $V_{E}$ affects the width of each segmentation area, and the value of $\alpha_{e}$ affects the attenuation rate of $E_{ij} (n)$. The smaller the $\alpha_{e}$, the higher the segmentation accuracy is. The two values can be adjusted by Eqs. (1) and (2).

1 $$\alpha_{e} = {\raise0.7ex\hbox{$C$} \!\mathord{\left/ {\vphantom {C \mu }}\right.\kern-0pt} \!\lower0.7ex\hbox{$\mu $}}$$

2 $$V_{E} = 2\mu$$

where* C* is a constant, and $\mu$ is the average intensity value of the given slice.

The connection strength $\beta$ between the neighboring neurons: the value of $\beta$ indicates the degree of interaction between neurons in the neighborhood. The larger the value of $\beta$ is, makes the $U_{ij} \left( n \right)$ fluctuate more violently. According to Weber-Fechner law, when the stimulus rises, changes, or the intensity of the stimulus corresponds to the stimulus itself, the sensation will be triggered, and the amount of sensation of the stimulus follows a simple logarithmic rule [37]. The internal mechanism of the synchronous pulse phenomenon is derived from the influence of two different inputs on the internal activity items. The main factor is the connection strength $\beta$. The subjective sensation of the human eye is roughly logarithmically related to the intensity of the input signal, the $\beta$ also plays a similar role and can be simulated by (3).

3 $$\beta = a\ln (\mu )$$

where $a$ is an experimental constant.

The exponential decay time constant $\alpha_{u}$ of the $U_{ij}$ affects the distribution interval of the $U_{ij}$. The variance of the image reflects the overall structural characteristics of the image. $\alpha_{u}$ represents the degree of neuronal memory, and $\alpha_{u}$ should be different for different images. If the whole image is relatively flat, the variance is small, and the neuron should be easier to maintain the previous state. If the image contains many different blocks, the variance is large, and the neurons should be more difficult to maintain the previous state. The smaller the value of the $\alpha_{u}$, the wider the distribution range of the internal activity $U_{ij}$. Therefore, $\alpha_{u}$ is inversely proportional to the variance of the image, which can be described by the logarithmic function of the reciprocal of the variance of the input image:

4 $$\alpha_{u} = \log \left[ {1/{\text{var}} (I)} \right]$$

where ${\text{var}}$ represents the function of calculating variance, $I$ represents the input image pixel matrix.

The initial matrix $E(0)$ is usually set to the maximum value of the input pixel matrix. In general, the highest intensity part of the tooth CBCT image is the enamel, and the $U_{ij}$ must be compared with the maximum value of the dynamic threshold.

5 $$E(0) = \max (I)$$

**Fig. 4:**
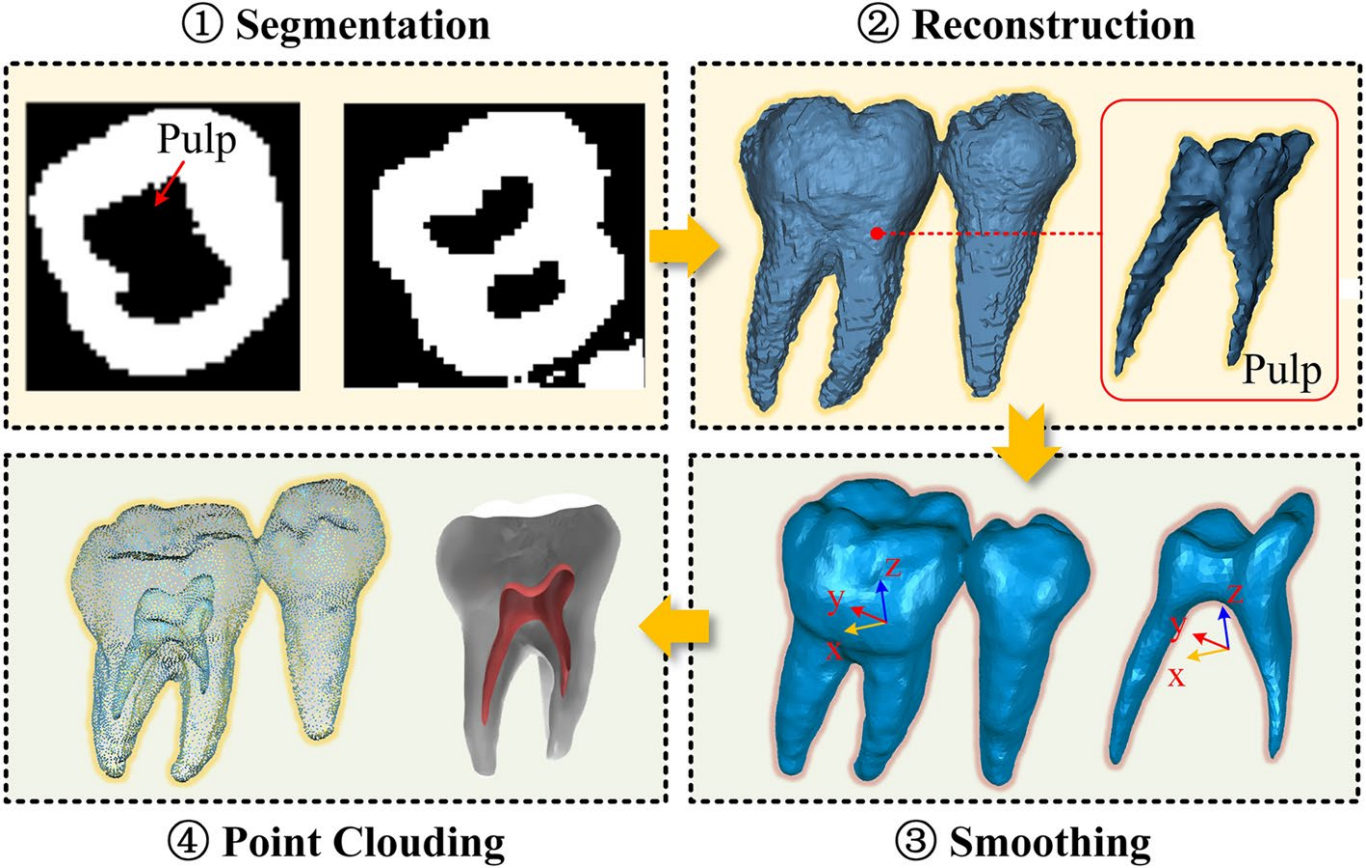
CBCT segmentation and pre-processing of target and neighboring teeth

Since the registration using a single tooth easily led to the local optimum, the segmentation of the mandibular molar (No. 36) as well as its neighboring teeth was performed using the adaptive SCM algorithm. The segmentation results are shown in Fig. 4. The segmented pulp and teeth were reconstructed using the marching cube method. Since the roughness of the model surface, we applied the Laplace smoothing method to the CBCT model and transformed it into point cloud data.

**Fig. 5:**
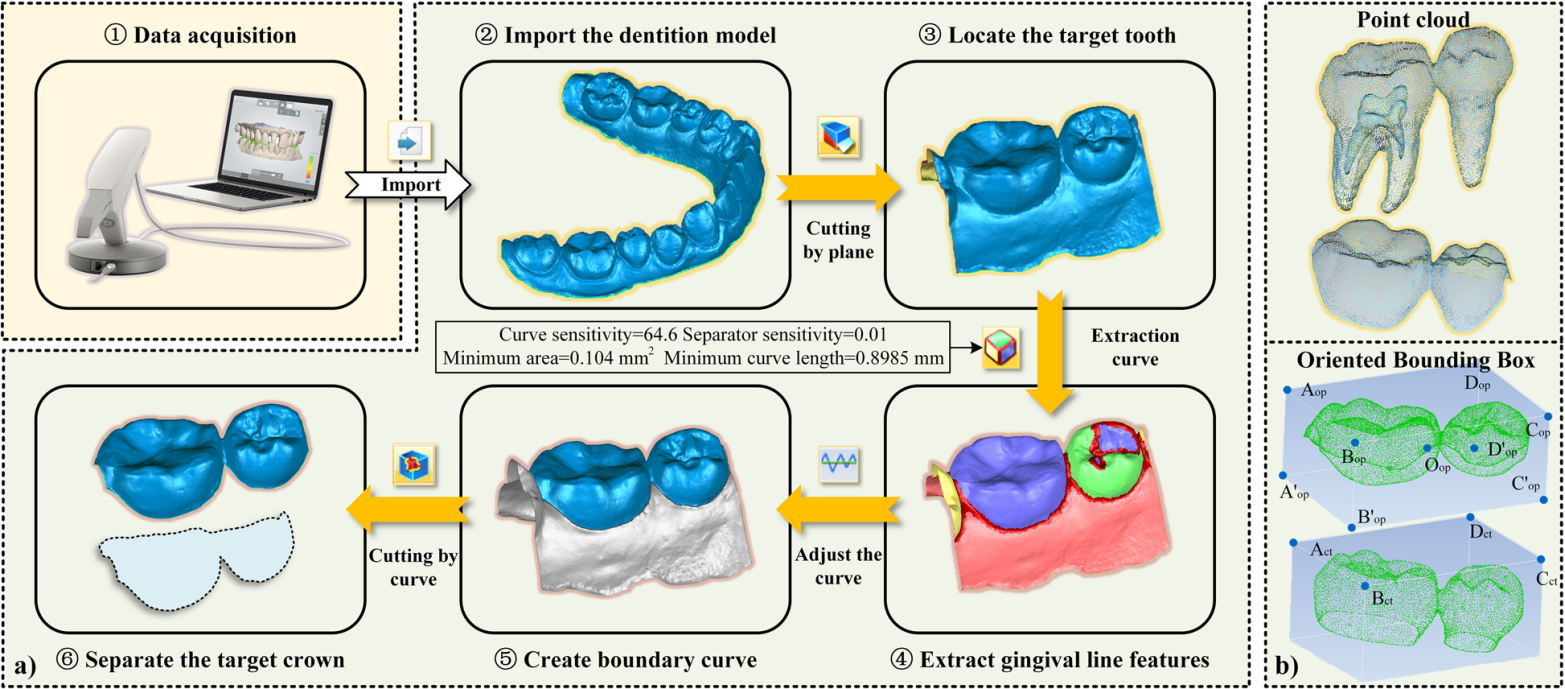
Model preprocessing. **a** Gingival margin line extraction and crown segmentation of optical scanned crown; **b** Equivalent processing of crown point cloud

As shown in Fig. 5a), the triangular mesh of the dentition was collected by an oral scanning device and imported into the “Geomagic wrap” software. We adjusted the curve generation coefficients according to the curvature distribution to extract the gingival margin curve. The gingival margin curve is used to segment the target crown mesh by “plane cutting”, “curve extraction”, “curve adjustment” and “curve cutting”. Considering the morphological differences between the two models, the Oriented Bounding Box (OBB) of the crown scanned model is established to intercept the equivalent crown CBCT model. Assuming that the coordinate of the point in the point cloud is $P_{i} (x_{i} ,y_{i} ,z_{i} )$, the center $O_{{{\text{op}}}}$ of the OBB is

6 $$O_{{{\text{op}}}} = \frac{1}{n}\sum\limits_{i = 1}^{n} {P_{i} }$$

Move the OBB center $${\text{O}}^{op}$$$$(o_{x}^{{{\text{op}}}} ,o_{y}^{{{\text{op}}}} ,o_{z}^{{{\text{op}}}} )$$ of scanned model to $$(o_{x}^{{{\text{ct}}}} ,o_{y}^{{{\text{ct}}}} ,o_{z}^{{{\text{op}}}} )$$, where $$o_{x}^{{{\text{ct}}}}$$、$$o_{x}^{{{\text{ct}}}}$$、$$o_{y}^{{{\text{op}}}}$$、$$o_{y}^{{{\text{ct}}}}$$、$$o_{z}^{{{\text{op}}}}$$ are OBB center coordinates of the scanned model and the CBCT model, respectively. The prongs $${\text{A}}_{{{\text{op}}}} {\text{D}}_{{{\text{op}}}}$$ were placed parallel to $${\text{A}}_{{{\text{ct}}}} {\text{D}}_{{{\text{ct}}}}$$ and $${\text{A}}_{{{\text{op}}}} {\text{B}}_{{{\text{op}}}}$$ parallel to $${\text{A}}_{{{\text{ct}}}} {\text{B}}_{{{\text{ct}}}}$$.The OBB of CBCT model was intercepted using the bottom surface $$A{'}_{op}B{'}_{op}C{'}_{op}D{'}_{op}$$ We retained the crown of tooth CBCT model, as shown in Fig. 5b).

### Registration method

#### Group 1: registration using the PHMSR method

**Fig. 6:**
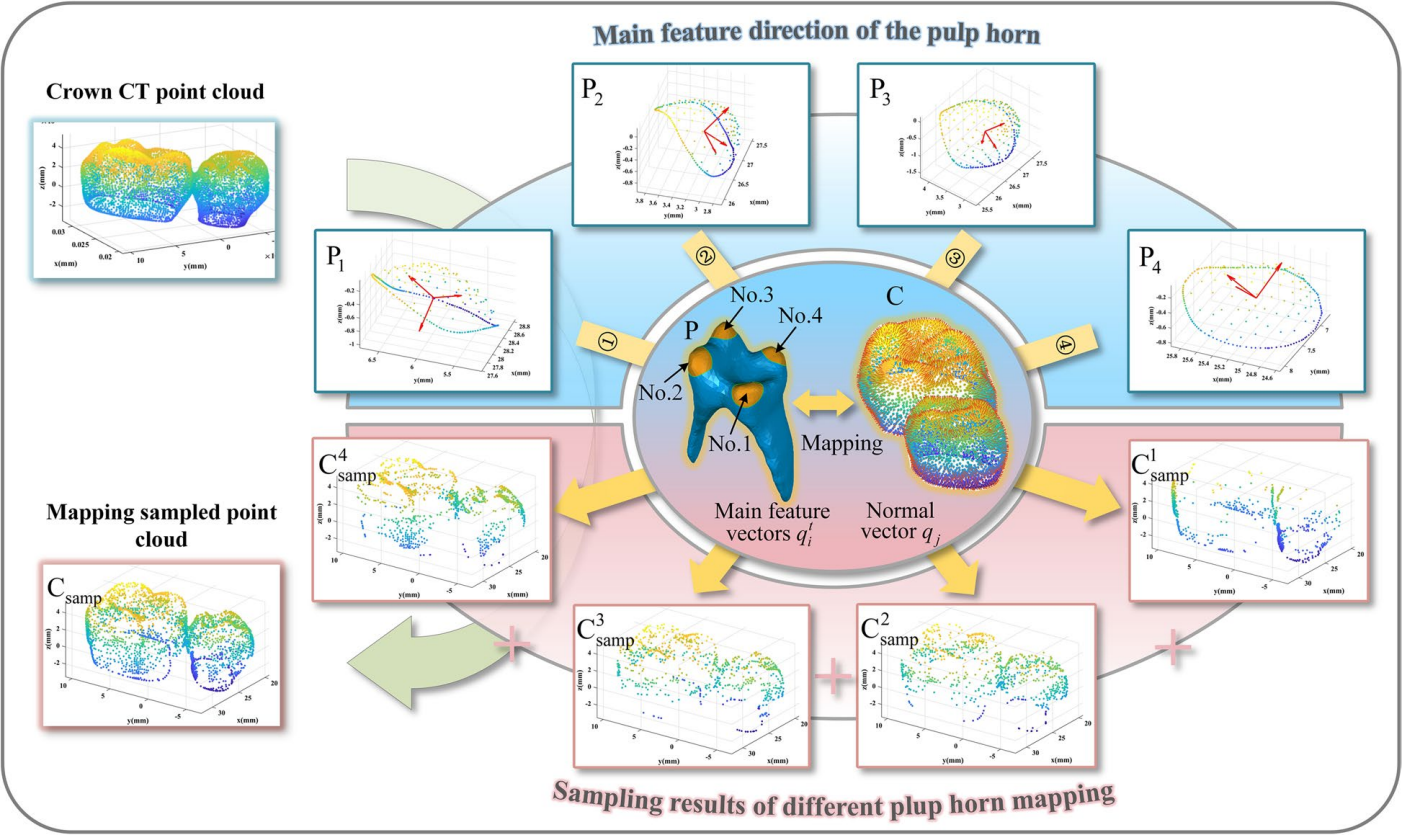
Feature directions of different pulp horns and their mapping results

Since the crown morphology of CBCT and scanned model is not consistent, and a lack of reasonable matching rules, the two types of point clouds cannot be directly matched. Hence, we identified the point set in the crown CBCT point cloud that affects the pulp pose by feature mapping algorithm, increasing the weight of them as well as the neighboring points in the crown registration, and the crown-pulp matching performed by the two crowns registration. The pulp horn is part of the intramedullary extension of the tooth tip into a cusp, and its shape and position are highly similar to the tooth cusp. It is opposite the top of the pulp chamber, which is the closest part to the occlusal surface. The pulp horn is the key feature to determine the pose of the whole pulp cavity. We were inspired by the normal space sampling algorithm [38], and the principle of the feature mapping algorithm is described as follows:

Calculating the feature direction of each pulp horn, estimating the normal vector of the crown surface. The angle between the normal vector and the feature direction of the pulp horn is taken as the mapping indicator, and retaining the points in the crown point cloud with greater correlation with the pulp horn feature. Record the indexes of these points, and surface reconstruction is performed around these points during the registration process. The pulp horn feature mapping (PHFM) algorithm is shown in Algorithm 1. Under different initial deviations between crown models, we can adjust the upper limit $\gamma_{{{\text{max}}}}$ to change the number of mapping points. The mapping diagram is shown in Fig. 6.

**Figure:**
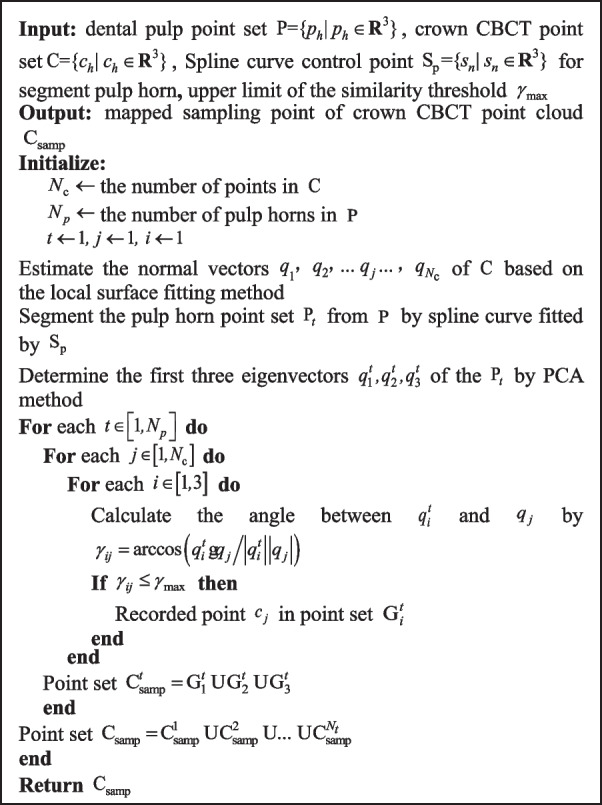
** Algorithm 1.** The PHFM for crown CBCT point cloud

Most of the teeth requiring RCT have caries, and the occlusal surfaces are usually of incomplete and non-smooth morphology. Since the noise and artifacts exist in the actual CBCT images, the slices in the occlusal region are discontinuous [39], and CBCT reconstructed point clouds of the tooth crown surface may produce discontinuous or outlying points with less surface accuracy than optical scanned models. The fine registration should not only be robust to point cloud registration with noise, outliers, and error points, but also use the feature surface as the reference unit of registration, and take into account the pulp pose.

We introduced the point-surface registration method. The basic principle is to calculate the surface patches formed by the corresponding neighborhoods of mapped points on the crown CBCT point cloud, and transform the point-point matching into point-surface matching. To robustly find the matching point pair between the point and surface, the surface is downsampled to obtain dense points. The influence of outliers and noise is reduced by adjusting the surface reconstruction parameters, and the sparse ICP algorithm is used to solve the transformation parameters of the newly established matching point pairs to ensure the final registration accuracy.

**Fig. 7:**
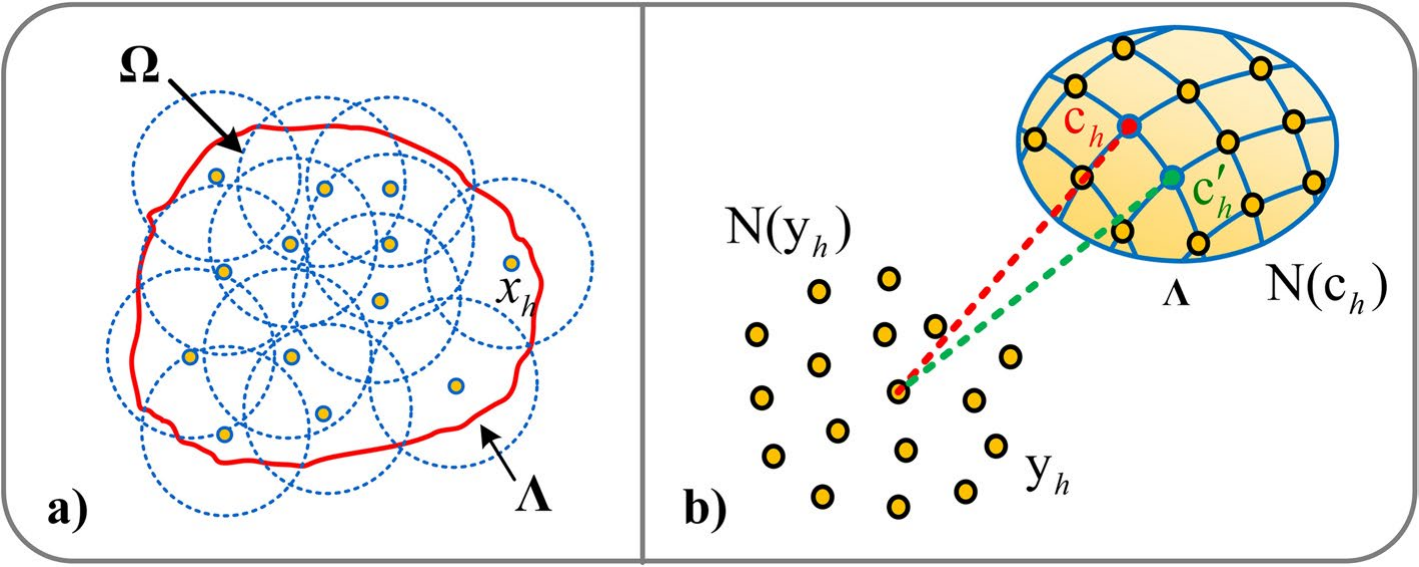
Registration algorithm. **a** MLS-based surface reconstruction principle; **b** Point-surface registration rules

To obtain more efficient matching point pairs, we used the quadric surface based on moving least squares (MLS) [40] as a reference surface (Fig. 7a). Compared with implicit surfaces such as the Poisson surface and Bessel surface, the quadric surface reconstruction speed is faster, and the MLS method can improve the local accuracy, and avoid the process of block fitting and smoothing.

The matching rules are as follows: As shown in Fig. 7b), Using crown CBCT point set $${\text{C}}$$ as a fixed object and optical scanned point set Y as a free object, the nearest point $$c_{h}$$ of $${\text{y}}_{h}$$ in $${\text{C}}$$ is determined. Given that the $$k_{y}$$-nearest neighbor point set of $${\text{y}}_{h}$$ is $${\text{N(y}}_{h} {)}$$, the $$k_{c}$$$$(k_{c} \ge 3k_{y} )$$-nearest neighbor point set of $$c_{h}$$ is $${\text{N(y}}_{h} {)}$$, which is set to the sample of local surface $$\Lambda$$ reconstruction. We can obtain the nearest point $$c^{\prime}_{h}$$ from point $$y_{h}$$ to surface $$\Lambda$$. Taking point $$c^{\prime}_{h}$$ as the new matching point of $${\text{y}}_{h}$$, that is, $${(}c_{h}^{\prime } {\text{, y}}_{h} {)}$$ is a new matching point pair. The algorithm is shown in Algorithm 2.

**Figure:**
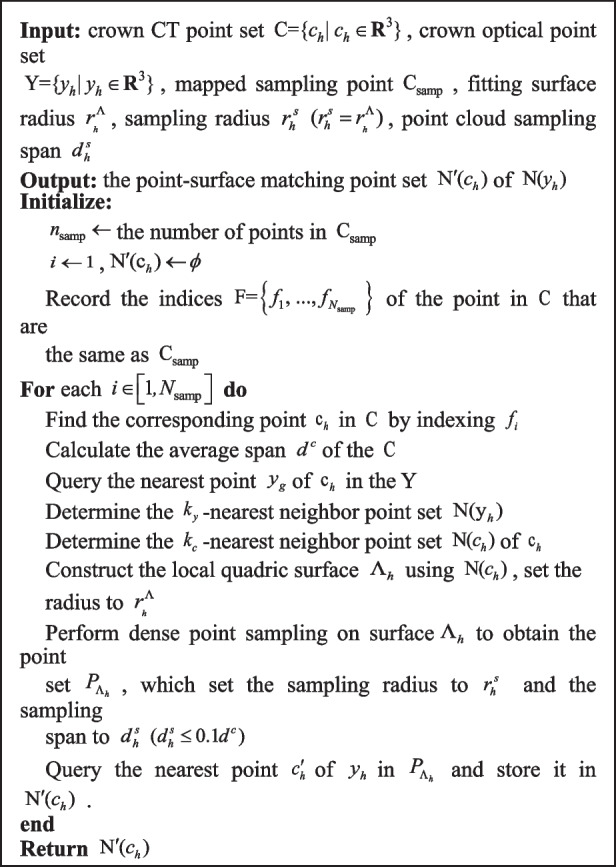
** Algorithm 2.** Search for matching point pairs between points and mapping surfaces

In this algorithm, the $r_{{_{h} }}^{\Lambda }$ can adjust the range of the point cloud used for the surface fitting, $r_{h}^{s}$ can adjust the range of dense sampling points on the surface $\Lambda_{h}$, specifically as follows:

- When the initial deviation between the free and the fixed point cloud is large, the $r_{{_{h} }}^{\Lambda }$ and the $r_{h}^{s}$ should be increased, expanding the matching range of the surface $\Lambda$ to ensure that $y_{h}$ finds the correct matching point.
- When the initial deviation between them is small, the $r_{{_{h} }}^{\Lambda }$ and $r_{h}^{s}$ can be reduced, then the point-surface matching degenerates to the point-point matching, to improve the calculation efficiency.
- When there are noises in the point clouds to be registered, we can select a suitable surface fitting radius $r_{{_{h} }}^{\Lambda }$ to filter the noise points and generate surfaces on the target point cloud, which improves the robustness of the alignment algorithm. In theory, the sampling span $d_{h}^{s}$ depends on the average span of the target point cloud and the accuracy requirements. Since the $d_{h}^{s}$ is too small will lead to a sudden increase in the amount of computation. Therefore, the selection of the parameter should take into account the hardware conditions and select the maximum value under the premise of satisfying registration accuracy.

**Fig. 8:**
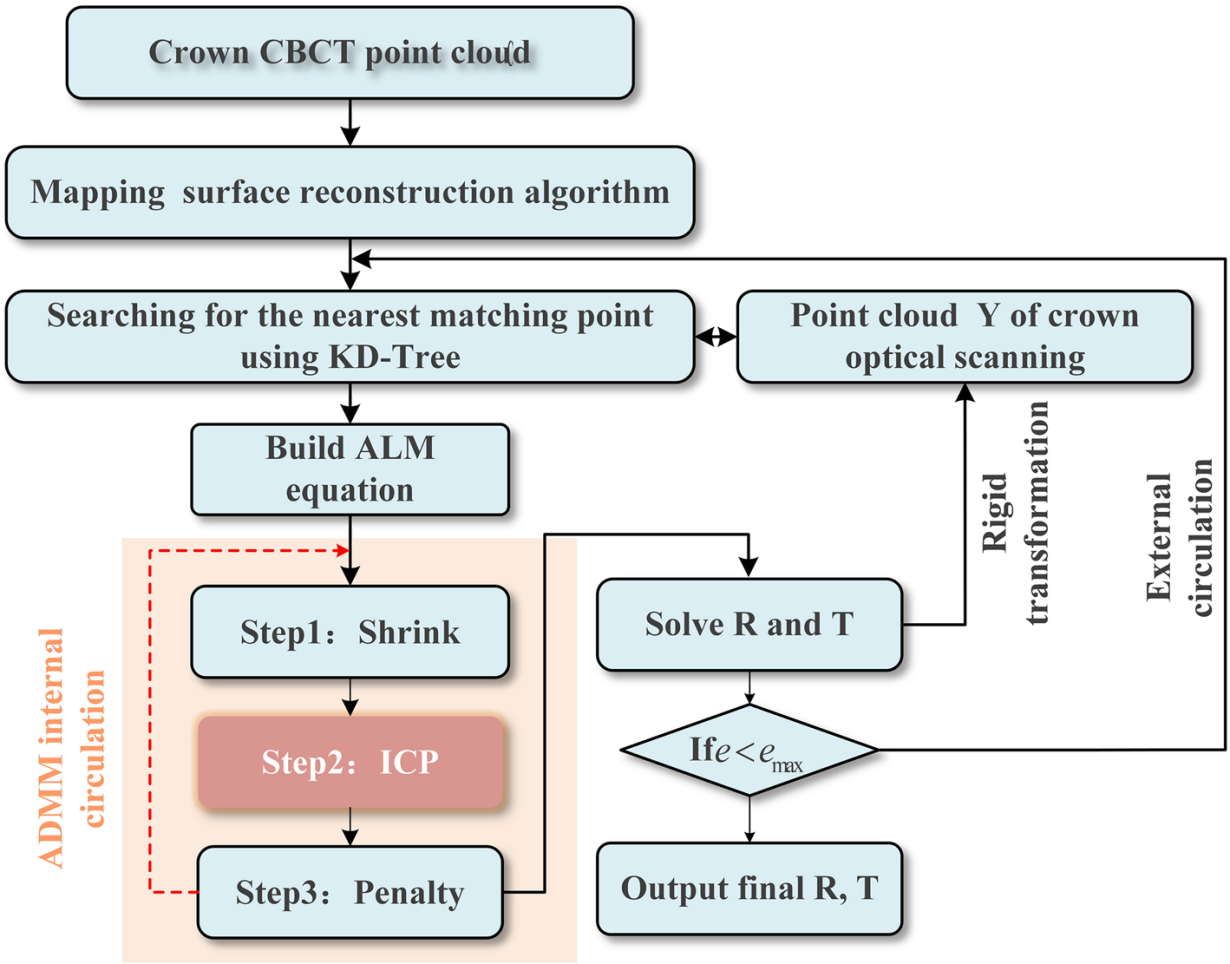
Flow chart of PHMSR registration algorithm

Based on the point-surface registration rules, the sparse ICP [31] is introduced into the registration process to enhance the robustness of the registration. The sparse ICP algorithm can be expressed by Eq. (7):

7 $$\left\{ {{\mathbf{R}}, \, {\mathbf{t}}} \right\} = \mathop {\arg \min }\limits_{{{\mathbf{R,t}}}} \sum\limits_{h = 1}^{n} {w^{p - 2} \left\| {{\mathbf{R}}c_{h}^{\prime } + {\mathbf{t}} - y_{h} } \right\|_{2}^{2} } + I_{SO(k)} ({\mathbf{R}})$$

where ${\mathbf{R}}$ and ${\mathbf{t}}$ are rotation and translation matrices respectively. The intermediate variable ${\mathbf{Z}} = \left\{ {{\mathbf{z}}_{h} \in R^{3} , \, h = 1, \, ..., \, n} \right\}$ is introduced to ensure a stable solution:

8 $$\left\{ {{\mathbf{R}},{\mathbf{t}}} \right\} = \mathop {\arg \min }\limits_{{{\mathbf{R}},{\mathbf{t}},Z}} \sum\limits_{h = 1}^{n} {\left\| {{\mathbf{z}}_{h} } \right\|_{2}^{p} } + I_{SO(k)} ({\mathbf{R}}){\text{ s}}{\text{.t }}{{\varvec{\updelta}}}_{h} = {\mathbf{0}}$$

where ${{\varvec{\updelta}}}_{h} = {\mathbf{R}}c_{h} + {\mathbf{t}} - {\text{y}}_{h} - {\mathbf{z}}_{h}$, the augmented Lagrangian method is an effective tool for solving the above-constrained optimization problems. The Eq. 9 can be defined as:

9 $$L_{A} ({\mathbf{R}},{\mathbf{t}},{\mathbf{Z}},{{\varvec{\Lambda}}}) = \sum\limits_{h = 1}^{n} {\left\| {{\mathbf{z}}_{h} } \right\|_{2}^{p} } + {{\varvec{\uplambda}}}_{h}^{T} {{\varvec{\updelta}}}_{h} + \frac{\mu }{2}\left\| {{{\varvec{\updelta}}}_{h} } \right\|_{2}^{2} + I_{SO(k)} ({\mathbf{R}})$$

where ${{\varvec{\Lambda}}} = \left\{ {{{\varvec{\uplambda}}}_{h} \in {\mathbb{R}}^{k} ,h = 1...n} \right\}$ is a set of Lagrange multipliers and $\mu (\mu > 0)$ is the penalty weight. Equation (9) contains four unknowns: rotation matrix ${\mathbf{R}}$, translation matrix ${\mathbf{t}}$, intermediate variable ${\mathbf{z}}$ and coefficient vector ${{\varvec{\Lambda}}}$. The alternating multiplier method (ADMM) is used to optimize this function. The PHMSR registration process is shown in Fig. 8. During the solution process, the matching target point in Step 2 is changed to the intermediate point $\tilde{y}_{i}$. Through the point-surface matching rule, the closer matching target point and intermediate point are found, which reduces the number of iterations to a certain extent and increases the accuracy.

The above process is programmed in a C +  + environment. In this paper, the $\gamma_{{{\text{max}}}}$ is set to 20° for small initial deviations, and 25° for large initial deviations. The $r_{{_{h} }}^{\Lambda }$ $(r_{{_{h} }}^{\Lambda } = r_{h}^{s} )$ is set to 0.1 for small initial deviations and 0.3 for large initial deviations. The parameters of sparse ICP are $p = 0.5$, $\mu = 10$, the iteration times of Step 2 is $n_{2} = 2$, and the number of convergences for step (2.1) is $n_{s} = 3$.

#### Group 2: registration using the Sparse ICP method

The source code of Sparse ICP method is from the Reference [31] and is implemented programmatically in a C++ environment. The parameters are set to $p=0.5$, $\mu=10$, the iteration times of Step 2 is $n_{2}=2$ , and the number of convergences for step (2.1) is $n_{s}=3$.

#### Group 3: registration using the ICP method

The source code of ICP method is from the Reference [14] and is implemented programmatically in a C++ environment. The maximum number of iterations is set to 100 and the allowable error is 0.1 mm.

#### Group 4: registration using the CPD method

The source code of the CPD method is from the Reference [32] and is implemented programmatically in a C++ environment.

**Fig. 9:**
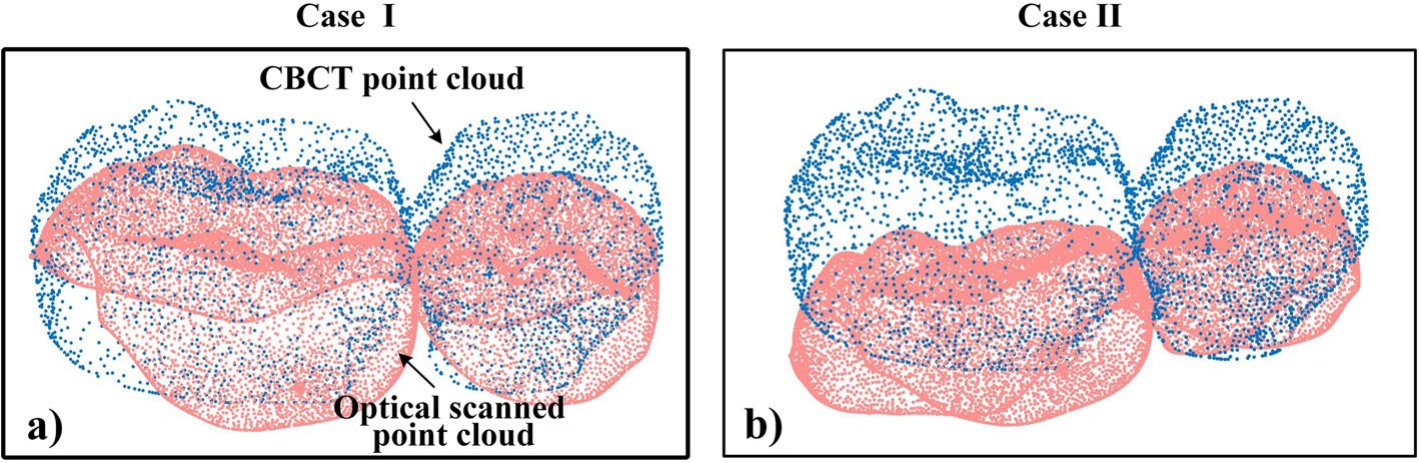
Relative pose of the point cloud. **a** Small initial deviation. **b** Large initial deviation

For each method, two different initial poses (large and small deviation) were set for the same group of crown point clouds for registration, as shown in Fig. 9, the crown CBCT point cloud as a fixed object and the optical scanned point set as a free object, twice registration results for one subject were averaged for evaluation.

#### Measurement evaluation

The accuracy of the registration was measured using three metrics: Root mean square error (RMSE) between the crown scanned and CBCT crown point cloud, crown-pulp orientation deviation (CPOD), and crown-pulp position deviation (CPPD), and are calculated using Matlab. When the RMSE is smaller, it indicates that the registration accuracy of the crown surface point cloud is higher; when the CPOD and CPPD are smaller, it indicates that the registration accuracy of the crown-pulp point cloud is higher.

The Root mean square (RSME) is commonly used to evaluate the point cloud alignment accuracy [41]. The measurement is formulated by:

10 $${\text{RSME}} = \sqrt {\frac{{\sum\limits_{h = 1}^{n} {(c_{h} - y_{h} )^{2} } }}{n}}$$

where $c_{h}$ denotes the point coordinates in the target point cloud (CBCT), and $y_{h}$ denotes the $k_{c}$-nearest neighbor point of $c_{h}$ in the source point cloud (optical scanned).

**Fig. 10:**
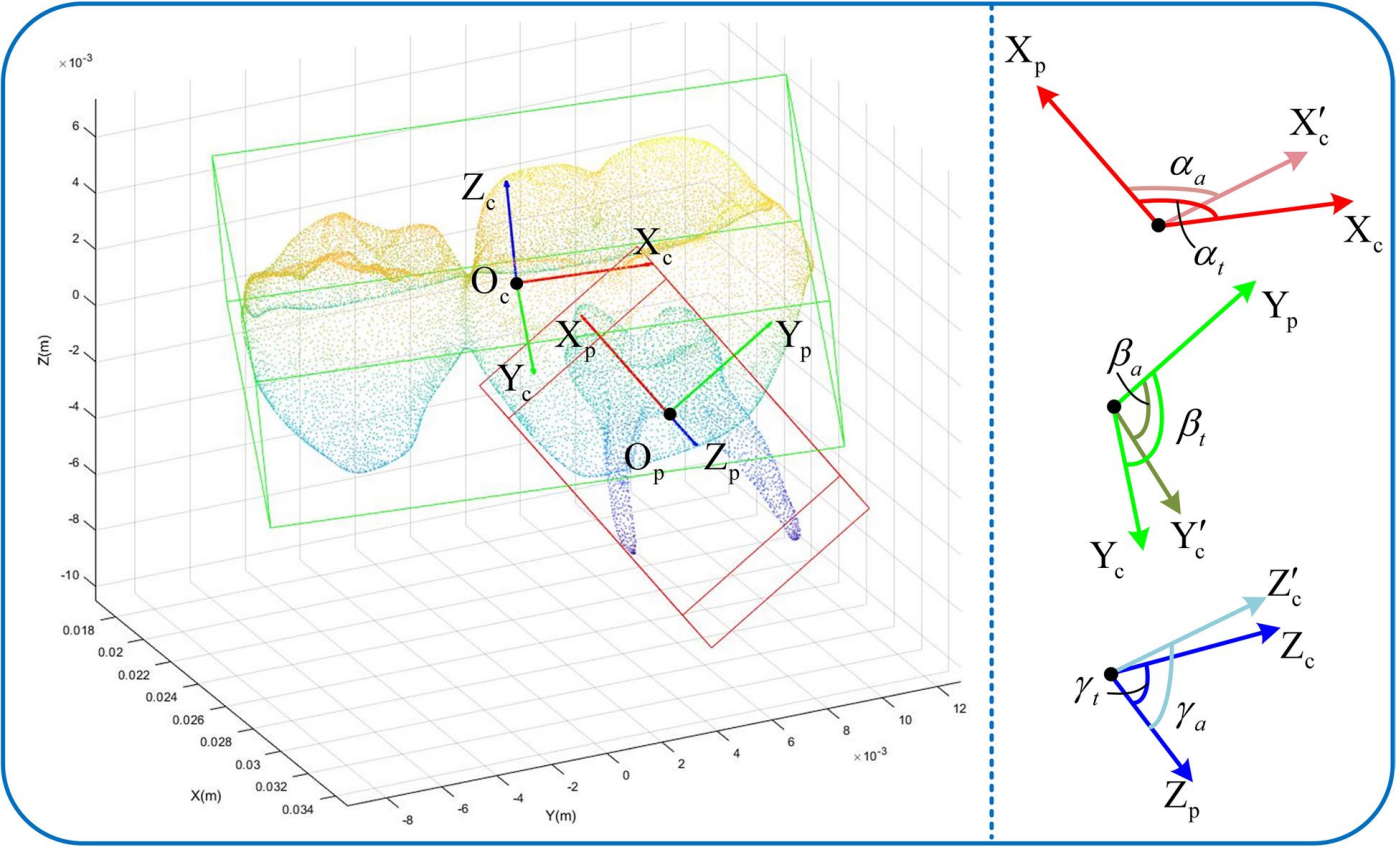
Evaluation of crown-pulp pose deviation

Since there are no relevant metrics regarding crown-pulp registration, we used the relative pose deviation of the optical scanned crown from the pulp CBCT point cloud as an evaluation measurement. The theoretical crown-pulp registration poses as a ground truth, then the crown-pulp Orientation deviation (CPOD) and position deviation (CPPD) were presented. As shown in Fig. 10, we used the PCA algorithm to calculate the centers of the correct pose crown and pulp point clouds, i.e., ${\text{O}}_{{\text{p}}} (x_{{\text{p}}} ,y_{{\text{p}}} ,z_{{\text{p}}} ) \,$ and ${\text{O}}_{{\text{c}}} (x_{{\text{c}}} ,y_{{\text{c}}} ,z_{{\text{c}}} ) \,$, then used them as the origin to establish theoretical coordinate systems, ${\text{O}}_{{\text{c}}} {\text{ - X}}_{{\text{c}}} {\text{Y}}_{{\text{c}}} {\text{Z}}_{{\text{c}}}$ and ${\text{O}}_{{\text{p}}} {\text{ - X}}_{{\text{p}}} {\text{Y}}_{{\text{p}}} {\text{Z}}_{{\text{p}}}$. Established the actual origin $O{'}_{c}(x{'}_{c},\,y{'}_{c},\,z{'}_{c})$ and coordinate system $O{'}_{c}-X{'}_{c},\,Y{'}_{c},\,Z{'}_{c}$ of the aligned post-crown. The measurements are formulated by:

11 $${\text{CPPD}} { = }\sqrt {(x_{a} - x_{t} )^{2} + (y_{a} - y_{t} )^{2} + (z_{a} - z_{t} )^{2} }$$

12 $${\text{CPOD}} { = }\sqrt {(\alpha_{a} - \alpha_{t} )^{2} + (\beta_{a} - \beta_{t} )^{2} + (\gamma_{a} - \gamma_{t} )^{2} }$$

where $x_{a} = x_{{\text{p}}} - x_{{\text{c}}}$, $y_{a} = y_{{\text{p}}} - y_{{\text{c}}}$, $z_{a} = z_{{\text{p}}} - z_{{\text{c}}}$, $\alpha_{a}$, $\beta_{a}$, and $\gamma_{a}$ are the position and orientation deviations of the registered crown scanned point cloud and the pulp CBCT point cloud in the *xyz* direction. $x_{t} = x_{{\text{p}}} - x^{\prime}_{{\text{c}}}$, $y_{t} = y_{{\text{p}}} - y^{\prime}_{{\text{c}}}$, $z_{t} = z_{{\text{p}}} - z^{\prime}_{{\text{c}}}$, $\alpha_{t}$, $\beta_{t}$, and $\gamma_{t}$ are the theoretical position and orientation deviations of the crown scanned point cloud and the pulp CBCT point cloud.

#### Statistical analyses

Statistical analyses were performed using SPSS software. Mean, standard deviation, and 95% confidence intervals were calculated for the errors of registration for the eight groups data. A two-tailed paired* t*-test was used to assess significant differences between each method at *p* < 0.05.

## Results

**Fig. 11:**
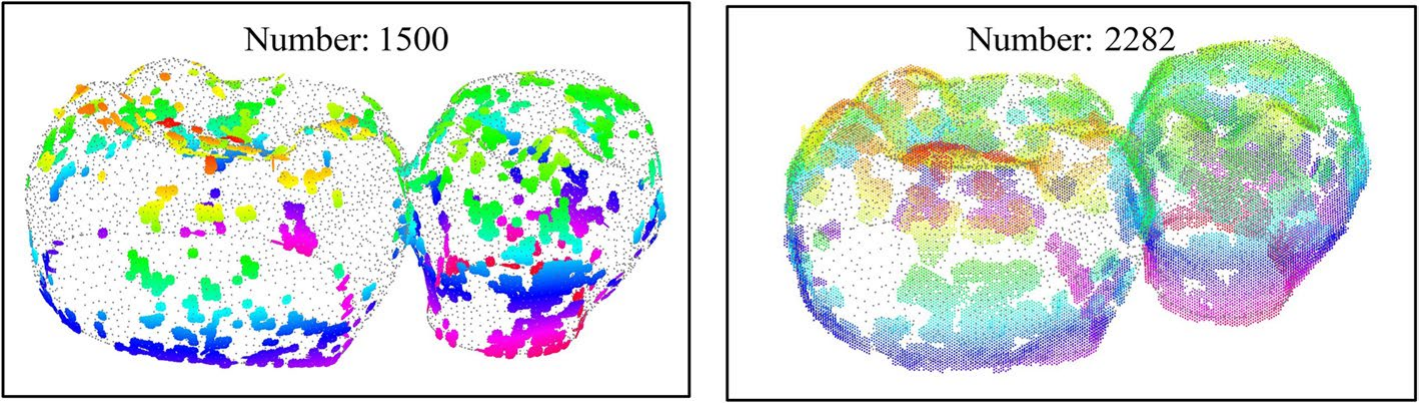
Surface reconstruction results of the PHMSR method in Case I and Case II

In the vitro experiments, CBCT and oral scanned models of eight groups (No. 35 and 36 teeth) were registered. As shown in Fig. 11, small surfaces were generated near the mapping points of the CBCT crown point cloud during the PHMSR registration process. When the initial deviation is small (Case I), fewer mapping surfaces and smaller radius are used to reduce the calculation time to reconstruct the surface. When the initial deviation is large (Case II), the number of mapping surfaces and the radius are enlarged to avoid local optimality of the matching and ensure the convergence of the crown registration.

**Fig. 12:**
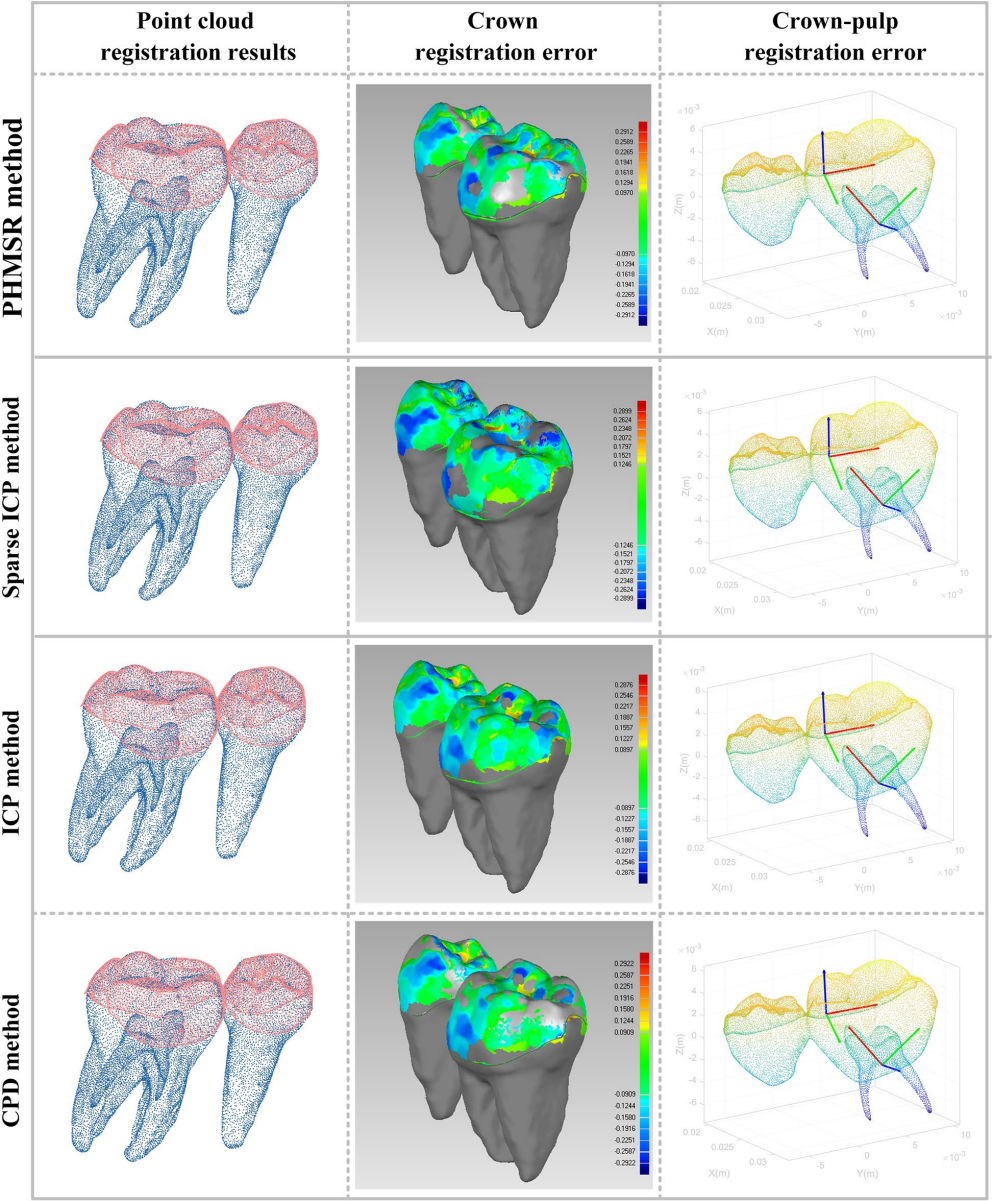
The registration results of four methods. A darker color in the color deviation chart indicates a larger registration error in these areas

As shown in Fig. 12, the crown surface deviation of the four methods did not differ much, and the range of error is approximated. With the pulp attitude unchanged, it is evident that the scanned crowns have a significantly different attitude and position. As shown in Table 1, according to the PHMSR method, the mean CPOD and CPPD deviations were 0.536° ± 0.295° and 0.064 ± 0.055 mm, respectively, which showed that the crown-pulp registration accuracies were significantly superior to the other conventional method. The difference of CPOD error between PHMSR group and other groups was statistically significant (*p* < 0.05), and the difference of CPPD error between PHMSR group, CPD group and ICP group was statistically significant.

**Table 1:** Statistical analysis of registration accuracy of different methods

Method	CPOD ± SD/°	CPPD ± SD /mm	RSME ± SD /mm	Duration ± SD [s]
PHMSR	0.536 ± 0.295 (0.289 ~ 0.783)	0.064 ± 0.055 (0.018 ~ 0.110)	0.250 ± 0.014 (0.238 ~ 0.261)	8.244 ± 2.885
Sparse ICP	4.089 ± 3.6175^*^ (2.535 ~ 5.630)	0.133 ± 0.114 (0.038 ~ 0.228)	0.257 ± 0.0170 (0.243 ~ 0.271)	0.013 ± 0.002
Sparse ICP-PHMSR	*p* = 0.0001	*p* = 0.1458	*p* = 0.3411	-
ICP	1.787 ± 0.767^*^ (1.146 ~ 2.428)	0.700 ± 0.318^*^ (0.434 ~ 0.966)	0.217 ± 0.0465 (0.178 ~ 0.256)	0.217 ± 0.021
ICP-PHMSR	(*p* = 0.0007)	(*p* = 0.0007)	(*p* = 0.7985)	-
CPD	1.665 ± 0.667^*^ (1.107 ~ 2.223)	0.718 ± 0.178^*^ (0.569 ~ 0.867)	0.209 ± 0.041 (0.175 ~ 0.243)	45.680 ± 20.220
CPD-PHMSR	(*p* = 0.0006)	(*p* = 0.0000)	(*p* = 0.0193)	-

*indicates that there is a significant difference between the accuracy metrics of the method and the PHMSR method, i.e., *p* < 0.05

**Fig. 13:**
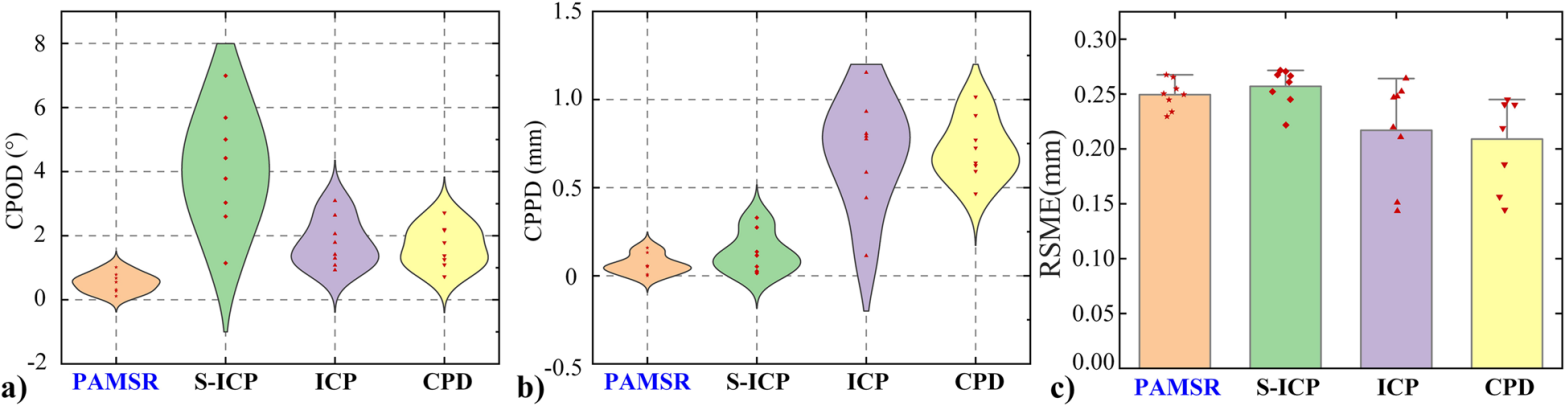
Comparison of registration errors of four methods. **a** CPOD registration error.** b** CPPD registration error. **c** RSME registration error

As shown in Fig. 13a), the CPOD of the PHMSR, Sparse ICP, ICP, and CPD method are distributed in the ranges of 0.289 to 0.783°, 2.535 to 5.630°, 2.535 to 5.630°, and 1.107 to 2.223°. The CPOD distribution interval of Sparse ICP is the largest, and the maximum error reaches 6.992°, indicating the poor stability of the method. The PHMSR method had the smallest error distribution interval and the maximum error was 1.017°. The maximum CPOD errors of ICP and CPD were close to each other with 3.092° and 2.7146°, respectively. As shown in Fig. 13b), the CPPD of PHMSR, Sparse ICP, ICP, and CPD were distributed in 0.018 ~ 0.110 mm, 0.038 ~ 0.228 mm, 0.434 ~ 0.966 mm and 0.569 ~ 0.867 mm. The distribution interval of the CPPD for ICP was the largest, and the maximum deviation of the ICP and CPD were 0.806 mm and 0.725 mm, respectively. In summary, the crown-pulp registration error distribution of PHMSR method is more centralized.

As shown in Fig. 13b) and Table 1, The PHMSR, Sparse ICP, ICP, and CPD method with error ranges of 0.238 ~ 0.261 mm, 0.243 ~ 0.271 mm, 0.178 ~ 0.256 mm, and 0.175 ~ 0.243 mm, respectively. The RSME of PHMSR and Sparse ICP is similar, and the RSME error of PHMSR was slightly larger than that of the ICP and CPD methods, but there was no significant difference in the accuracy of the crown registration with Sparse ICP (*p* = 0.3411) and ICP (*p* = 0.7985).

In terms of computational time, the registration time of PHMSR is more than that of the sparse ICP and ICP methods, but less than that of the CPD method.

## Discussion

PHMSR algorithm is an important innovation in this paper. By finding the mapping relationship between pulp horn and crown, small surfaces are reconstructed near the mapping point of the crown CBCT point cloud to increase the accuracy of crown-pulp registration, and propose the corresponding accuracy indexes, which solves the problem of poor applicability of the generalized method in dental crown-pulp registration. Since the point cloud of the optical scanned and the CBCT reconstruction point cloud are not exactly the same in morphology, number, and density, the optimal registration is probably not point-to-point, and the traditional methods use the original corresponding points for the registration, thus the registration error of the crown surface is small, and the crown-pulp pose deviation is large. For our proposed method, thanks to the reconstruction of the mapping surface, the target points matching the points in the source point cloud are newly created point pairs, and thus the registration error of the crown surface is slightly larger than the other three methods, but the crown-pulp relative pose is adequately taken into account. In addition, the stability of the PHMSR algorithm (SD(CPOD) = 0.295° and SD(CPPD) = 0.055 mm) was significantly higher than that of Sparse ICP, ICP, and CPD algorithms.

The registration is the digital preparation before pulp opening, the registration deviation directly affects the final positioning result. From the clinical studies [15–22], it can be found that the surface registration errors in the maxillary and mandibular ranged from 0.13 to 0.345 mm, which means that the RSME deviations (0.1434 to 0.2295 mm) of the four methods in our study are acceptable in the clinic, and the reasons for the discrepancies may include the differences in the quality of the CBCT, the accuracy of the segmentation, and the accuracy of the oral scanned model. Taking the mean root deviation (0.1678 ± 0.3178 mm) before and after registration reported by Lee et al. [17] as a reference, it can be seen that the deviation of the crown-pulp position (0.064 ± 0.055 mm) of the PHMSR method is perfectly acceptable. At present, there is no research on crown-pulp registration, therefore, the relationship between the use of digital technology to assist root canal opening and registration accuracy is discussed. Zehnder et al. studied the digital guide-assisted root canal treatment of upper anterior teeth. The distance deviation was 0 ~ 1.59 mm, and the angle deviation was 0 ~ 5.60° [42]. The mean apical deviation obtained by Jacobs et al. [8] was 0.10 ~ 1.88 mm, and the angle deviation was 0.18 ~ 5.87°. The distance deviation of the drill needle obtained by Zhang et al. [9] is 0.183 ~ 0.537 mm, and the angle deviation is 1.770° ~ 3.260°. The mean distance deviation of the apical obtained by Torres et al. [10] was 0.63 ± 0.35 mm, and the mean angle deviation was 2.81° ± 1.53°. Taking study [10] as an example, the mean crown-pulp registration deviation (0.536° and 0.064 mm) of PHMSR accounted for 10.16% of the distance deviation and 19.07% of the angle deviation. The crown-pulp orientation deviation (4.089°) of Sparse ICP exceeded the surgical deviation range, and the position deviation (0.133 mm) accounted for 21.11% of the surgical deviation. The crown-pulp deviation of ICP (1.787°) accounted for 63.59% of the surgical deviation, and the crown-pulp deviation of CPD (1.665°) accounted for 59.25% of the surgical angle deviation. Their position deviation exceeded the range of surgical distance deviation. It can be seen that our PHMSR method can have the least impact on surgical planning, thus ensuring the accuracy of surgery.

During the registration, the PHMSR method needs to traverse the neighboring points of the mapped point cloud to fit the new surface and then densely downsample the surface, this process is computationally intensive, resulting in a longer time compared to sparse ICP and ICP, but the time cost is still much smaller than the CPD method.

Although it is the first implementation that the pulp pose is indirectly introduced into the crown registration, there are some limitations to our study: First, the sample size of the study was only 8 cases, and all of them were young people in China, whose dental pulp had not been reduced or calcified. In addition, we mainly used molar teeth for the study, which have four pulp horns. For the teeth with anatomical variation and pulp injury, the feature direction of the pulp horn may be difficult to reflect the information of pulp pose, so the clinical applicability of the algorithm needs to be further studied. Secondly, PHMSR method can be regarded as an improved version of sparse ICP, including parameters such as the number of mapping points and the radius of surface reconstruction. Parameter tuning and the selection of pulp horn still requires manual interaction. Therefore, the automatic parameter setting method will be further studied in the future.

## Conclusions

Within the limitations of this vitro study, it was concluded that the PHMSR method has a smaller crown-pulp registration accuracy and a clinically acceptable deviation range than conventional methods, which may be used for clinical virtual planning of inlay-guided root canal therapy, although further clinical studies are needed to confirm our results.

## Data Availability

The datasets used and analysed during the current study are available from the corresponding author on reasonable request.

## Abbreviations

CBCT: Cone Beam Computed Tomography
3D: Three-Dimensional
PHMSR: Registration method based on pulp horn mapping surface
2D: Two-Dimensional
STL: Standard tessellation language
DICOM: Digital imaging and communications in medicine
SCM: Spiking cortical model
RMSE: Root mean square error
CPD: Coherent point drift
MLS: Moving least squares
ICP: Iterative closest point
CPOD: Crown-pulp orientation deviation
CPPD: Crown-pulp position deviation
PCL: Point cloud library
